# Circumcision in hemophilia using low quantity of factor concentrates: experience from Dakar, Senegal

**DOI:** 10.1186/s12878-017-0080-1

**Published:** 2017-04-24

**Authors:** Moussa Seck, Aloïse Sagna, Mame Sokhna Guéye, Blaise Félix Faye, Diariétou Sy, Sokhna Aissatou Touré, Abibatou Sall, Awa Oumar Touré, Saliou Diop

**Affiliations:** 1 Hematology Service, Cheikh Anta Diop University, Dakar, BP 5005 Senegal; 2 Pediatric Surgical Service, Cheikh Anta Diop University, Dakar, BP 5005 Senegal; 3 Hematology Service, National Blood Transfusion Center, Dakar, BP 5005 Senegal

**Keywords:** Hemophilia A, Circumcision, Bleeding complications

## Abstract

**Background:**

Circumcision in hemophiliacs is a delicate surgery because of bleeding risks that could be avoided by adequate substitution of coagulation factor. This practice is very challenging in countries where anti hemophilic treatment is inaccessible. The study aimed to evaluate a circumcision protocol in hemophilia A using low quantities of factor concentrates.

**Methods:**

This prospective study included 26 hemophiliacs A who underwent circumcision in 2014. Medical treatment protocol using low quantity of factor concentrates was drafted by physicians of the Hemophilia Treatment Center and the surgical protocol by experienced surgeons. Assessment criteria were: number of hospitalization days, number of exposure days to factor concentrates, delay to healing and occurrence of bleeding events.

**Results:**

Mean age was 9.6 years (1–30). Hemophiliacs patients were classified as severe (*n* = 8), moderate (*n* = 9) and mild form (*n* = 9). Mean number of exposure days to factor VIII concentrates was 6.9 days (5–12) in children and 10.75 days (7–16) in adults (*p* = 0.0049); mean number of hospitalization days was 3.68 days (2–10) in children and 13.5 days (13–15) in adults (*p* = 0.0000); delay to healing was 26.47 days (20–35) in children and 25.25 days (22–30) in adults (*p* = 0.697); five haemophiliacs (19.2%) presented bleeding events after the circumcision. The mean amount of FIII concentrates used per patient was 1743 IU (810–2340).

**Conclusion:**

The study shows treatment protocol using low quantity of factor concentrates is efficient in hemophilia patients who underwent circumcision.

## Background

Hemophilia is an inherited bleeding disorder related to sex (*F8* and *F9* gene are located on X chromosome), due to a deficiency of FVIII (hemophilia A) or FIX (hemophilia B) clotting factors. Hemophilia causes internal bleeding (hematoma, hemarthrosis) or spontaneous external bleeding or following minor trauma or surgery [1, 2].

In Africa, hemophilia is often discovered during circumcision that is the first surgery in young healthy boy and consisting of foreskin removal [3].

There are many and various indications for circumcision according to the different culture and civilization [4–6]. In some countries, none circumcision is a very difficult resented for both hemophiliacs and their parents who consider circumcision practice as an act of great religious value and social recognition for the young boy [7, 8]. On the other hand, none circumcision installs a social inferiority complex; so that despite the increased risk of excessive bleeding that can be fatal, hemophiliacs want to be circumcised as well as healthy subjects [9–11].

Nevertheless its realization constitutes a real controversy in the world especially if there are no medical indications [12, 13].

Circumcision in hemophilia often requires the use of large quantities of clotting factor concentrates to ensure proper hemostasis. For these reasons, some haemophiliacs can’t be circumcised in countries where factor concentrated are unavailable or not easily accessible. This has led some authors to implement substitutive protocols using low quantities of anti hemophilic factor [14–16].

In Senegal, circumcision was performed in 61.4% of followed hemophiliacs’ patients and was the first circumstance of diagnosis in 18% of hemophiliacs [17]. Currently, 179 hemophiliacs are regularly followed at Dakar’s hemophilia treatment center and mean per capita Factor VIII and IX use in 2014 was 0.033 IU in Senegal while the World Federation of Hemophilia recommends 1 IU per capita to ensure the needs of hemophiliacs [18].

In this context, the use of low amounts of factors remains an option for the treatment of hemophilia. For this reason we have evaluated this approach in the context of circumcision of hemophilia patients.

## Methods

We included 26 haemophiliacs A with or without inhibitor and who underwent circumcision in 2014. Diagnosis of hemophilia is performed on the basis of prolonged aPTT associated with FVIII levels less than 30% (1). Data collected at the beginning of the study are presented in Table 1. Hemophiliacs were monitored regularly at Dakar’s hemophilia treatment center every 4 months. They are received in emergency when they have bleeding.

**Table 1:** Characteristics of study population

Parameters	Number (%)
Age (mean age: 9.6 (1–30 years)	
≤15 years	22 (84.6%)
>15 years	4 (15.4%)
Severity of hemophilia	
Severe	8 (30.8%)
Moderate	9 (34.6%)
Mild	9 (34.6%)
Treatment history by factor VIII concentrates	
Patients treated	25 (96%)
Patients untreated	1 (4%)
Number of exposure days to factor VIII concentrates	
1–10 days	20 (76%)
11–20 days	2 (8%)
21–30 days	4 (16%)
Anti factor VIII inhibitors	
Patients with inhibitors	4 (15.3%)
Patients without inhibitors	22 (84.7%)

An inhibitor screening was done when the number of days of exposure to FVIII concentrates reaches 30 days by the Bethesda method. The tests were performed using reagents from Diagnostica Stago (Asnieres, France). None of haemophiliacs had prophylactic treatment.

All uncircumcised hemophiliacs since 2010 were registered and selection of patients was done according to their order of listing. There was no selection criteria based on age, severity of hemophilia, the presence or absence of inhibitors.

This prospective study was conducted at Dakar’s Hemophilia Treatment Center where hemophiliacs are followed. Data were registered in the medical chart, including for each haemophiliac, the treatment history by FVIII concentrates and cumulative number of exposure days to anti hemophilic treatment. Circumcision was realized in the pediatric surgery department of Albert Royer Hospital for infants and in another medical center for adults.

Haemophiliacs and their parents were informed of the organization of a circumcision session through the Senegalese Association of Hemophiliacs. An informed consent form has been signed by patients or their parent if they are under 18 years old.

### Medical treatment protocol

The substitutive treatment by FVIII concentrates was as follow: each patient had received 30 IU/kg of FVIII concentrates one hour before surgery. This dose was repeated systematically every 24 h for 48 h. An additional dose was given during occurrence of a persistent bleeding despite compressive dressing with tranexamic acid. Recombinant FVIII concentrates (Refacto or Recombinate) were used for haemophiliacs without anti FVIII inhibitors and bypassing factors such as factor eight inhibitor bypass activity (FEIBA) or activated recombinant factor VII (rFVIIa) for haemophiliacs with inhibitors. FEIBA was used at 80 IU/kg for the first three doses and rFVIIa at a dose of 90 μg/kg every 3 h and intravenous bolus when hemophiliacs had bleeding.

Tranexamic acid (Exacyl®) was administered at 20 mg/kg every 12 h by direct intravenously for three days, and local application during dressings.

Oxacillin (Bristopen®) was administered orally of 50 mg/kg/day for 10 days. An analgesic level one (paracetamol) was administered orally of 15 mg/kg every 6 h in children and 500 mg to 1 g every 8 h for adult if required.

### Surgery protocol

Haemophiliac is placed in dorsal recumbency position. After skin disinfection with iodine polyvydone, the pubic symphysis is identified and traction is exerted on the penis.

Local anesthesia is carried out with lidocaïne 1% with a total dose of 2 mg/Kg observed before 5 min after injection. The injection is administered either side of the median line, into under pubis space at 10 and 2 o’clock compared to the root of the penis halfway between the summit of the two folds drew on the penis and lower edge of pubis.

This penile block is completed by subcutaneous anesthesia wreath at the base of the penis. After capping and carefully cleaning the smegma, surgeon carries out an identification of the level of skin section up to the base of the glans through the Kocher clamp. This is then placed perpendicularly to the shaft of the penis after having assured the absence of the glans by bidigital palpation.

A circular cross section of the skin is made at a cold blade then hemostasis using electrocautery is made. The Kocher clamp is placed after dissection at the level of mucosal sheet about 2 mm below the level of skin section and then cut at the cold blade.

The surgeon carries out an additional hemostasis using electrocautery followed by dermo-epidermal sutures using Vicryl wire. After soapy polyvydone toilet and drying with ether, surgeon applies a compressive bandage for 24 h with a swab containing iodized polyvydone wrapped around the glans. Removal of the dressing was made at the first postoperative day. The sore is left open to the air and eosin 2% was applied twice a day.

### Clinical evaluation

The outcome measures were: number of hospital days, number of exposure days to FVIII concentrates, healing time and occurrence of bleeding events.

Healing time is defined by the period from the beginning of circumcision to healing. Bleeding event is any haemorrhage that occurs since the release of hemophiliacs in the operating room until healed.

Externally, hemophiliacs were consulted at the hemophilia treatment center twice a week for two weeks then once a week until healed. Control of inhibitors was done in all hemophiliacs after healing.

### Statistical analysis

Data collection was done according to a survey sheet. Data was entered into the computer with Epi info version 6 and analyzed with statistical analysis system (SAS). Descriptive analysis was realized with a quantitative study and a qualitative study. Bivariate analysis was carried out by comparing the proportion of two qualitative variables using chi-square test and the comparison of the means by Student’s *t* test. These tests were performed according to their condition of applicability and significance p (probability of error) was fixed: *p* ≤ 0.05.

## Results

### Characteristics of the study population

There were 26 patients with hemophilia A who underwent circumcision included in this study.

Median age was 7.5 years (range 1–30 years); 4 haemophiliacs (15.3%) were aged over 20 years. According to the severity, 8 haemophiliacs (30.8%) had a severe form, 9 patients (34.6%) had a moderate form and 9 patients (34.6%) had a mild form.

Twenty five patients (96%) had been previously treated with FVIII concentrates but only six patients (24%) had more than 10 exposure days to FVIII concentrates.

Four patients (15.3%) had anti FVIII inhibitors. They had low titer inhibitor between 1.5 and 3.8 BU/ml (Table 1).

### Clinical evaluation

Mean number of days of FVIII concentrates treatment was 6.9 days (5–12 days) in children and 10.75 days (7–16 days) in adults (*p* = 0.0049). Mean number of days of hospitalization was 3.68 days (2–10 days) in children and 13.5 days (13–15 days) in adults (*P* < 0.001). Mean healing time was 26.47 days (20–35 days) in children and 25.25 days (22–30 days) in adults (*p* = 0.697).

Bleeding events occurred among five haemophiliacs (19.2%). Bleeding occurred spontaneously in one patient who had anti factor VIII inhibitors or during dressings in four patients. In patients without inhibitors, tranexamic acid helped to stop bleeding while in patients with inhibitor, rFVIIa was used to stop bleeding.

We noted that severe haemophiliacs were most exposed to treatment with a higher number of days of hospitalization and a prolonged healing time. Similarly, they had more bleeding events (Table 2).

**Table 2:** Clinical evaluation according to hemophilia severity

Severity	Severe form	Others forms (*n* = 18)		*p*
	(*n* = 8)	Moderate (*n* = 9)	Mild (*n* = 9)	
Days of hospitalization (3–15 days)				
3–10 days	2 (25%)	8 (88.9%)	2 (22.2%)	0.04
11–15 days	6 (75%)	1 (11.1%)	7 (77.8%)	
Days of treatment (5–16 days)				
5–10 days	3 (37.5%)	7 (77.8%)	8 (88.8%)	0.01
11–16 days	5 (62.5%)	2 (22.2%)	1 (11.2%)	
Delay to healing (20–45 days)				
20–30 days	6 (75%)	7 (77.8%)	9 (100%)	0.09
31–45 days	2 (25%)	2 (22.2%)	0	
Hemorrhagic complications (*n* = 5)				
Patients with complications	4 (50%)	1 (11.1%)	0	0.03
Patients without complications	4 (50%)	8 (88.9%)	9 (100%)	

### Characteristics of haemophiliacs with bleeding events

Five haemophiliacs (19.2%) presented bleeding events. This was four severe haemophiliacs and one moderate haemophiliac. Among them, three severe haemophiliacs had developed anti FVIII inhibitors. Mean age of these patients was 15.2 years (6–30 years). Mean number of days of hospitalization was 11.2 days (6–15 days) and mean number of days of treatment was 9.2 days (5–14 days). Mean healing time was 28 days (22–35 days) (Table 3).

**Table 3:** Characteristics of haemophiliacs with bleeding complications

Patients	Age (years)	Severity	Inhibitors (BU/L)	Hospitalization (days)	Treatment (days)	Healing (days)	Rescue treatments
1	6	Severe	3.8	10	8	28	rFVIIa
2	6	Severe	3	10	7	25	rFVIIa
3	26	Severe	1.5	15	14	35	rFVIIa
4	30	Severe	0	15	12	30	Refacto
5	8	Moderate	0	6	5	22	Refacto

### Number of doses and total quantity of factor concentrates administered

Total quantity of FVIII concentrates administered was 38350 IU, soit 58.1 IU/kg per patient. Mean dose per patient was 1743 IU (810–2340); 12 haemophiliacs received Refacto (21160 IU) and 10 haemophiliacs received Recombinate (17190 IU) (Table 4)

**Table 4:** Number of doses and total quantity of factor concentrates administered

Hemophiliacs severity	Bleeding	Inhibitors	Factors concentrates	Doses	Total
1, severe	Yes	Yes	FEIBA/rFVIIa	3/10	3600 IU/450 μg
2, severe	Yes	Yes	FEIBA/rFVIIa	3/6	10800 IU/810 μg
3, severe	Yes	Yes	FEIBA/rFVIIa	3/5	5280 IU/330 μg
4, severe	Yes	No	Refacto	10	7800 IU
5, severe	No	No	Refacto	5	1050 IU
6, severe	No	No	Refacto	5	900 IU
7, severe	No	No	Refacto	5	1500 IU
8, severe	No	Yes	FEIBA/rFVIIa	3/2	2640 IU/66 μg
9, moderate	Yes	No	Refacto	5	1800 IU
10, moderate	No	No	Refacto	3	810 IU
11, moderate	No	No	Refacto	3	1440 IU
12, moderate	No	No	Refacto	3	1080 IU
13, moderate	No	No	Refacto	3	1170 IU
14, moderate	No	No	Refacto	3	1170 IU
15, moderate	No	No	Refacto	3	1270 IU
16, moderate	No	No	Refacto	3	1170 IU
17, moderate	No	No	Recombinate	3	900 IU
18, mild	No	No	Recombinate	3	1440 IU
19, mild	No	No	Recombinate	3	1170 IU
20, mild	No	No	Recombinate	3	1620 IU
21, mild	No	No	Recombinate	3	1710 IU
22, mild	No	No	Recombinate	3	1890 IU
23, mild	No	No	Recombinate	3	2070 IU
24, mild	No	No	Recombinate	3	2340 IU
25, mild	No	No	Recombinate	3	2340 IU
26, mild	No	No	Recombinate	3	1710 IU

Total quantity of FEIBA administered was 22320 IU and mean dose per patient was 5580 IU (2640–10800).

Total quantity of rFVIIa was 1656 μg and mean dose per patient was 414 μg (66–810). All haemophiliacs with inhibitors received FEIBA during the first three doses followed by rFVIIa in case of bleeding.

## Discussion

Circumcision in hemophilia is a delicate surgery because of bleeding risks. These bleedings can be prevented through proper supplementation of clotting factor concentrates, explaining the challenges in countries where substitutive treatment is not always available. It is demonstrated that using high quantity of factor concentrates in surgery in hemophilia is associated with inhibitor development whose management is more difficult because of using more costly treatments [19].

This study used low quantity of anti FVIII concentrates associated with antifibrinolytic treatment. It aims to reduce the risk of severe bleeding and the risk of developing anti FVIII inhibitors. Substitution therapy in our study consisted to raise FVIII levels around 60% for three days. An additional dose levels is systematically performed in serious bleeding events resistant to local hemostasis with tranexamic acid.

All hemophiliacs received only three doses systematically for increasing the factor level to 60% for three days. The amount of factor concentrates used in our study is less important than those used in Turkish and Moroccan studies [14, 15].

The effectiveness of this protocol was evaluated by comparing our results with those of circumcision in non-hemophiliac population. The criteria of comparison were based on the number of days of exposure to anti FVIII concentrates, the number of hospital days, the healing delay and bleeding complications.

The prolonged healing time in our patients may be explained by the high number of hemophiliacs aged over 15 years. More shortened recovery periods have been described in some studies but these studies were performed in hemophiliacs children aged less than 10 years [20].

Bleeding complications were noted in 19.2% of patients. This result is comparable to hemorrhagic complications of circumcision performed in healthy subjects whose prevalence of bleeding events is estimated between 10 and 35% [21, 22].

Many types of alternative protocols was used in various studies [14, 23, 24]. Al these protocols have the same objectives to reduce or prevent bleeding events of circumcision. These studies show that the absence or decrease in risk of bleeding was not linked to the use of high doses of anti FVIII concentrates but to the number of exposure days to antihemophilic treatment. All studies that have associated tranexamic acid and fibrin glue to strengthen local hemostasis results to a less occurrence of bleeding events [14, 24, 25]. Reduction of bleeding complications during hemophiliac’s circumcision is not only related to the use of large quantities of anti hemophilic factor. Other factors including antifibrinolytic treatment, good local hemostasis using electrocautery and the practice of circumcision by an experienced surgeon [26–28] are also implicated. The nursing staff must be qualified in the field of hemophilia management because most bleeding is post traumatic during the dressing [29].

Other surgical devices are newly used for proper local hemostasis such as diathermic knife which has reduced the need for anti FVIII treatment and the cost of circumcision [30].

According to age, it is proven that circumcision performed in newborns and children is easier, less grave and causes fewer complications compared to adults [31] whereas healing is more difficult and prolonged than in adults [32]. This can explain why circumcision is rarely practiced in adults except in hemophilia where circumcision is delayed if the anti hemophilic treatment is inaccessible.

Severe Hemophilia is associated with more morbidity with an upsurge in bleeding complications and a significant risk of inhibitor development. Hemophiliacs with inhibitors had more bleeding complications and a longer period to healing [33, 34].

## Conclusion

We show through this study that it is possible to realize circumcision in haemophiliacs in a country with low resources, using low quantities of anti hemophilic factor concentrates. This protocol is adaptable to a country where hemophilia treatment is not available permanently.
